# The Weekend Drug; Recreational Use of Sildenafil Citrate and Concomitant Factors: A Cross-Sectional Study

**DOI:** 10.3389/fmed.2021.665247

**Published:** 2021-08-20

**Authors:** Berhanemeskel Weldegerima Atsbeha, Beza Tefera Kebede, Biruktawit Shewafera Birhanu, Dawit Kumilachew Yimenu, Wudneh Simegn Belay, Chilot Abiyu Demeke

**Affiliations:** ^1^Department of Pharmaceutics and Social Pharmacy, College of Medicine and Health Sciences, University of Gondar, Gondar, Ethiopia; ^2^College of Medicine and Health Sciences, University of Gondar, Gondar, Ethiopia

**Keywords:** community pharmacy, erectile dysfunction, PDE5Is, sildenafil, Viagra

## Abstract

**Background:** Men who use erectile dysfunction medications for recreational purposes may be at increased risk of becoming psychologically dependent, which in turn could lead to psychogenic-based erectile dysfunction symptoms. Sildenafil has become one of the most commonly prescribed and abused drugs available today.

**Objectives:** This study aimed to describe the utilization pattern and associated factors of sildenafil citrate among its users visiting community pharmacies in Gondar, Ethiopia.

**Methods:** A facility-based cross-sectional study was conducted from March 20, 2017 to May 10, 2017, among male clients who visited community pharmacies in Gondar town, North West Ethiopia. A self-administered, structured questionnaire was used to collect data from Sildenafil users older than 18 years, that started using the drug (Sildenafil) for at least 6 months before the data collection period. A regression analysis was conducted to determine the association between study variables, and a *P*-value of <0.05 was considered to declare statistical significance.

**Results:** A total of 65 men participated in the study. Of the total study participants, 33.8% were aged 25–34 years and about 40% of them had multiple sexual partners. The use of sildenafil for recreational purposes was 66.2% and was found to be higher than its use for medical purposes 33.8%. Use of the drug for <1-year duration (AOR = 34.086, 95% CI [2.90, 401.37]) and 2 years duration (AOR = 21.42: 95% CI [2.10, 218.82]) were significantly associated with its use for recreational purpose. Non-recreational use of sildenafil includes erectile problems associated with diabetes mellitus (27.1%), heart disease (9.2%), hyperlipidemia (4.2%), and relationship problems due to stress and poor communication (3.1%).

**Conclusion:** Most men who use sildenafil citrate do so for recreational purposes, and use of sildenafil citrate for 2 years or less was associated with recreational use. There should be a collaborative effort among pharmacists, health professionals, and policymakers to improve the rational use of sildenafil.

## Introduction

Erectile dysfunction (ED) can be defined as a failure to attain or maintain an erection adequate for pleasing sexual activity. It is a relatively common medical problem and is estimated to affect 322 million people worldwide, of whom 30 million live in Africa (1). Its etiology can be organic, psychogenic, or most commonly, derived from a combination of the two (2). ED is age-associated, with prevalence rates ranging from 5–9% for men 18–39 years, 11–18% for men 40–59 years, and 44–70% for men 60 years, and older (3).

Medical risk factors for ED include diabetes mellitus, cardiovascular conditions (e.g., hypertension, hypercholesterolemia, atherosclerosis, and a recent history of myocardial infarction), pituitary/gonadal dysfunction, anemia, and renal/hepatic failure. Psychogenic causes can include depression and anxiety. Besides, medications may be a causal factor in which ~25% of cases of ED are caused by either prescription or over-the-counter medications. Drugs used socially such as nicotine, alcohol (in excess), and other recreational drugs also increase the prevalence of organic ED (2).

Sildenafil, a selective inhibitor of phosphodiesterase type 5 (PDE5), is an effective oral medication for the treatment of erectile dysfunction. During sexual stimulation, the cavernous nerves release nitric oxide (NO), which induces cyclic guanosine monophosphate (cGMP) formation and smooth muscle relaxation in the corpus cavern sum. Sildenafil facilitates the erectile process during sexual stimulation by inhibiting PDE5 and thus blocking the breakdown of cGMP (4). Sildenafil has become one of the most commonly prescribed and used pharmaceuticals available today. In the U.S., the fastest-growing segment of users of Phosphodiesterase 5 inhibitors (PDE5Is) are males aged 18–45 years (5). Soon after sildenafil was launched in Great Britain, its recreational use was observed in 10% of young men (aged 19–34 years old) (6). Similar results were also reported in other areas (7).

A cross-sectional survey conducted in undergraduate institutions within the Unites States revealed that participants had recreationally used an Erectile Dysfunction Medication (EDM) at least once in their lives (4%), some with current use (2.2%) (8). The majority of recreational EDM users reported mixing EDMs with illicit drugs and particularly during risky sexual behaviors (8).

In the Ethiopian context, due to the lack of strong regulations, sildenafil can be easily accessed from pharmacies without a prescription (1, 9–11). A qualitative study conducted in Addis Ababa, Ethiopia revealed that there was a widespread use of sildenafil citrate and the main reasons for the utilization were to gain sexual confidence and suppress anxieties associated with a feeling of loss of manhood (12).

Sildenafil use has also been associated with various adverse effects especially owing to its vasodilator properties. Caution should be exercised in patients with one or more risk factors for cardiovascular or cerebrovascular disease, which include myocardial infarction, cardiac arrest, angina, ventricular tachycardia, hypertension, and other cardiac symptoms. Concomitant use with organic nitrates (e.g., glyceryl trinitrate, isosorbide dinitrate) has also resulted in cardiac deaths, possibly due to sildenafil potentiating the nitrates hypotensive effects (4, 5, 13).

More frequent EDM use was associated with decreased erectile confidence, which in turn showed negative relations with erectile function. Men who use EDMs for recreational purposes may be at increased risk of becoming psychologically dependent, which in turn could lead to psychogenic-based ED symptoms (14).

There are also case reports associating the use of Sildenafil with recurrent epistaxis. The number of similar cases might increase due to the diffusion of PDE-5 inhibitor overuse in recreational settings (15, 16). Case reports of type A and B aortic dissection have also been reported with sildenafil abuse (17). In some cases, patients may experience vomiting, diarrhea, and abdominal pain shortly after the ingestion of sildenafil and may be found to have aortic dissection upon admission to a hospital (18).

In Ethiopia, the sale of PDE5Is as over the counter is prohibited. It is allowed only in licensed pharmacies to be dispensed with legal prescriptions and even drug stores [Pharmacy retail outlets managed by pharmacy diploma holders (known as druggists in Ethiopia)] are not allowed to hold these drugs. However, the regulatory control is not stringent, and thus illegal drug marketing is very common which leads to difficulty in monitoring their utilization and improve their outcome (19, 20). Their easy accessibility may also predispose people who took other medications concomitantly and those with comorbid conditions to the adverse effects of these drugs (13, 21, 22). Therefore, the current study aimed to assess the utilization pattern and associated factors of sildenafil citrate among its users visiting community pharmacies in Gondar, Ethiopia.

## Methods

### Study Design, Period, and Setting

A facility-based cross-sectional study was conducted among male clients who visited community pharmacies in Gondar town, North West Ethiopia. Gondar is located 738 km away from Addis Ababa, the capital city of the country. At the time of the study, there were 20 pharmacies and 34 drug stores in the town. Based on a projection from the National Census conducted in 2007, its population was estimated to be around 249,851 with the male to female ratio estimated to be around 1.5:1 (23). The study was conducted from March 20, 2017 to May 10, 2017.

### Source Population

All men in Gondar town that use sildenafil citrate for sexual function.

### Study Population

All sildenafil citrate users in Gondar town that fulfilled the inclusion criteria.

### Sample Size and Sampling Technique

All sildenafil citrate users that fulfilled the inclusion criteria and that were available (visited the community pharmacies) during the study period were included and thus no sample size calculation was needed. All of the available community pharmacies in the town were approached, but only seven of them were volunteer to allow the data collectors to meet with patients (collect data). Drug stores were not included in the study as it is illegal for them to sell phosphodiesterase inhibitors (such as sildenafil) in Ethiopia, though there were rumors that claim these drugs are sold illegally in these outlets.

### Inclusion and Exclusion Criteria

#### Inclusion Criteria

All men sildenafil users of at least 18 years of age that were volunteer to participate in the study.

#### Exclusion Criteria

Sildenafil users who started using the drug (Sildenafil) for <6 months before the data collection period.

### Study Variables

The dependent variable was the use of sildenafil citrate for recreational purposes and this was analyzed against the independent variables; socio-demographic factors (age, marital status, partner status, educational status, occupation, religion) and other factors such as illicit substance use, and duration of sildenafil use.

### Data Collection Procedure

A structured, self-administered questionnaire was used to collect data. The data collection tool (questionnaire) was originally prepared in English, and to minimize any language bias/misunderstanding, it was translated to Amharic (the local language) and re-translated back to English to check for consistency in meaning. The questionnaire was composed of 36 questions that were divided into three major parts. The first part asked about the socio-demographic (age, marital status, partner status, educational status, occupation, religion…) and clinical characteristics of the study participants. The second and third parts were focused on the participant's general perception and utilization pattern of sildenafil citrate. The data was collected by two data collectors who were well aware of the questionnaire as well as the purpose of the study.

### Data Quality Assurance

The data collectors were given 1 day of training on the purpose of the study and the data collection procedures. A pretest was carried out on 5 sildenafil users that came to two randomly selected pharmacies in the town. Based on the results, modifications (removal of questions that were not feasible in the current setup and/or re-writing of the wordings in simpler forms) were applied to the questionnaire and those participants in the pretest were excluded from the final study. The collected data were checked for completeness and consistency each day before entering into the software for analysis.

### Data Processing and Management

Statistical analysis was conducted using IBM SPSS version 20. Descriptive analysis was conducted and results were presented using tables. Binary logistic and multi-variable logistic regression analyses were conducted to determine the association between the dependent and independent variables. Variables that were significant at a *P*-value of <0.05 in the binary logistic regression were selected for multivariable logistic regression and in the multivariable regression analysis, those variables that were significant at a *p*-value of <0.05 with a 95% confidence interval were regarded as significant factors that contribute to the recreational use of sildenafil citrate.

### Operational Definitions

**Recreational use:** Use of a drug to advance certain activity (sexual performance) without a definite need or any medical condition.

**Abuse:** Prolonged and/or excessive use of a drug (sildenafil).

### Ethical Consideration

The study protocol was approved by the Ethical Review Committee of the School of Pharmacy, University of Gondar, Ethiopia with an approval number of SoP 826/09. Before the data collection, the purpose of the study and the proceedings were provided to each study participant and verbal consent was obtained. No personal identifiers were included in the questionnaire and confidentiality of the information was assured in such a way that no disclosure of the collected data was made apart from the study purpose.

## Results

### Socio-Demographic Characteristics

A total of 65 men had participated in the study. The mean age of the participants was 37 years (range 22–67). More than half (52.3%) of the study participants were married and 41.5% of the participants had completed secondary education (Grade 9–12). Regarding occupational status, about 24 (36.9%) of the respondents were employees, and 42 (64.6%) were Orthodox Christians by religion (Table 1).

**Table 1:** Socio-demographic characters of sildenafil users in Gondar town, 2017.

**Variables**	**Category**	**Total (*N* and %)**	**Recreational use *N* (%)**	**Medical (by prescription) *N* (%)**	**Statistical test**
Age	18–24	12 (18.4)	10 (83.3)	2 (16.7)	X2 = 17.570, *P* < 0.001
	25–34	22 (33.8)	19 (86.3)	3 (13.7)	
	35–44	13 (20)	7 (53.8)	6 (46.2)	
	45–54	9 (13.8)	4 (44.4)	5 (55.6)	
	>54	9 (13.8)	6 (66.7)	3 (33.3)	
Marital status	Married	34 (52.3)	17 (50.0)	17 (50.0)	X2 = 8.308, *P* = 0.004
	Other (single, widowed)	31 (47.7)	5 (16.1)	26 (83.9)	
Partner status	One stable partner	39 (60.0)	19 (48.7)	20 (51.3)	X2 = 13.238, *P* < 0.001
	Occasional partner	26 (40.0)	24 (92.3)	2 (7.7)	
Educational status	Read and write	8 (12.3)	4 (50.0)	4 (50.0)	X2 = 5.040, *P* = 0.188
	Grade 1–8	7 (10.8)	3 (42.9)	4 (57.1)	
	Grade 9–12	29 (44.6)	23 (79.3)	6 (20.7)	
	College and university	21 (32.3)	13 (61.9)	8 (38.1)	
Occupation	Unemployed	9 (13.8)	8 (88.9)	1 (11.1)	X2 = 13.598, *P* = 0.006
	Employed	24 (36.9)	12 (50.0)	12 (50.0)	
	Retired	7 (10.8)	2 (28.6)	5 (71.4)	
	Student	8 (12.3)	8 (100.00)	0 (0.00)	
	Merchant	17 (26.2)	13 (76.5)	4 (23.5)	
Religion	Orthodox	42 (64.6)	25 (59.5)	17 (40.5)	X2 = 2.580, *P*= 0.250
	Muslim	16 (24.6)	12 (75.0)	4 (25.0)	
	Other (catholic, protestant)	7 (10.8)	6 (85.7)	1 (14.3)	

### Sexual Characteristics

The majority of the study participants (58.4%) reported that they have at least once experienced unsafe sexual practices. Thirty-nine (60%) of the participants had one stable partner while 26 (40%) had occasional or multiple partners. The respondents also reported the use of alcohol and multiple illicit drugs with sildenafil in which more than half of the respondents reported that they use alcohol (63.1%) followed by cigarettes (36.9%) and khat (23.1%).

### Sildenafil Utilization

Regarding the source of information about sildenafil, some of the respondents reported multiple sources though the main source for the majority of them were their friends (70.6%). Regarding the source of the medication itself (sildenafil) some of the respondents also obtained it through multiple routes with the majority (89.3%) reporting without a prescription [Over-The-Counter (OTC) basis]. In the current study, 43 participants (66.2%) had used sildenafil for recreational purposes (Table 2).

**Table 2:** Sildenafil utilization characteristics of users in Gondar town, 2017.

**Variable**		**Frequency**	**Percentage (%)**
Information source about the drug (sildenafil)	Healthcare professionals	15	23.1
	Friends	46	70.7
	Media	9	13.8
	Medication leaflet	8	12.3
	Family	2	3.1
The acquisition method of the drug	With prescription	15	23.1
	Without prescription	58	89.3
Reason for use	Recreational	43	66.2
	Medical (by prescription)	22	33.8
The main reason for recreational usage	To increase erectile sensation	40	61.5
	To increase rigidity	41	63.1
	Enhancing self-esteem	29	44.6
	To impress or satisfy a partner	17	26.2
	To decrease the refractory phase	11	16.9
	To treat erectile dysfunction	11	16.9

Multiple answers were also reported concerning the timing of taking the drug (sildenafil) in which 40 (61.5%) of them reported that they take it 30 min before having sex, 13 (20%) whenever they thought of having sex, and 12 (18.5%) use it immediately before sex. About 84.6% of the participants reported that the sildenafil price was affordable and 67.7% of the participants reported it is easily accessible in the community pharmacies.

In the duration of use, 33 (50.8%) of the users took sildenafil for less than or equal to 1 year, 18 (27.7%) for 2 years and 14 (21.5%) had taken it for more than 2 years. Most of the participants (84.5%) also reported that they have not encountered any negative pressure from society and 83.1% wished to use sildenafil again. Regarding information provision, 69.2% of the participants preferred it in written form, 33.8% through radio and TV, and 10.8% *via* audiovisual recordings. The most common side-effect experienced by the users due to the use of sildenafil was dizziness (69.2%) followed by headache (49.2%), blurred vision (29.2%), longer erection time (9.2%), and stomachache (4.6%).

### Factors Associated With Sildenafil Use

Binary and Multi-variable logistic regression analyses were conducted to determine if there was an association between the various independent variables and the recreational use of sildenafil by taking the medical use of sildenafil as constant. Duration of sildenafil use, partner status, marital status, and age were found to be significantly associated with the recreational use of sildenafil in the binary logistic regression analysis at *P* < 0.05. Those significant variables were then analyzed in the multivariable logistic regression. However, only duration of sildenafil use was significantly associated (at *P* < 0.05) with recreational use of the medication in which men who used sildenafil for <2 years were more likely to use it for recreational purposes compared with those chronic users who used it for >2 years. Those users that used the medication for <1 year were 34 times more likely to use it for recreational purposes (AOR = 34.086, 95% CI [2.90, 401.37]), while those users of 2 years duration were 21 times more likely to use it for recreational puro=pose as compared to more chronic users of >2 years (AOR = 21.42: 95% CI [2.10, 218.82]) (Table 3).

**Table 3:** Factors associated with recreational use of sildenafil in Gondar town, 2017.

**Variable**	**Recreational use of sildenafil**						
	**Yes (%)**	**No (%)**	**Crude odds ratio (COR)**	***P*-value**	**Adjusted odds ratio (AOR)**	***P*-value**	
Age	18–24	10 (15.3)	2 (3.1)	10.00 (1.280, 78.117)	0.028	0.001 (0.001, 2.923)	0.998
	25–34	19 (29.2)	3 (4.6)	12.67 (2.002, 80.142)	0.007	0.977 (0.040, 15.749)	0.989
	35–44	7 (10.8)	6 (9.2)	2.33 (0.400, 13.609)	0.346	0.472 (0.014, 12.590)	0.675
	45–54	4 (6.1)	5 (7.7)	1.60 (0.237, 10.809)	0.630	0.603 (0.029, 23.923)	0.744
	>54	6 (9.2)	3 (4.6)	0.50 (1)	0.327	1	1.000
Marital status	Married	16 (24.6%)	17 (26.1%)	0.17 (0.05, 0.56)	0.040	0.970 (0.10, 9.00)	0.977
	Other wise	27 (41.5%)	5 (7.7%)	5.40 (1)	0.030	1	1.000
Partner status	One stable	19 (29.2%)	20 (30.7%)	0.079 (0.016, 0.382)	0.002	0.123 (0.011, 1.388)	0.090
	Multiple	24 (36.9%)	2 (3.1%)	12 (1)	0.001	1	1.000
Duration of use	≤1 year	27 (41.5%)	6 (9.2%)	27.0 (4.75, 153.64)	<0.001	34.086 (2.90, 401.370)	0.005
	2 years	14 (21.5%)	4 (6.1%)	21 (3.26, 135.48)	0.001	21.420 (2.10, 218.82)	0.010
	>2 years	2 (3.1%)	12 (18.5%)	0.17 (1)	0.019	1	1.000

On the other hand, some of the users also took sildenafil for medical purposes in which diabetes mellitus (27.1%), heart disease (9.2%), hyperlipidemia (4.2%), and relationship problem due to stress and poor communication (3.1%) were the most common conditions.

## Discussion

In the current study, it was found that there was a widespread use of sildenafil citrate for non-medical (recreational) purposes in the younger community. The result was consistent with similar studies conducted in other countries in which the majority of the younger participants use PDE5Is for recreational purposes (1, 6). This could be convincing because ED is usually common in older age and chronic diseases such as hypertension and type II diabetes. PDE5Is reduce systemic and pulmonary arterial pressures and cardiac output thereby having vasodilator effects to increase blood flow to the penis to help a man get and keep an erection. However, these diseases are usually not common at a younger age and thus younger men will usually tend to use sildenafil to impress or satisfy their partner rather than treat ED.

In the present study, the recreational use of sildenafil was also more prevalent in participants who had multiple sexual partners. Similar findings were also reported in a study conducted in Brazil (6), and this could be because men with multiple sexual partners may be attempting to impress their partners with prolonged sexual periods (7, 8). It could also be attributed to a low level of self-esteem in men who had multiple sexual partners.

In the current study, most of the participants reported that they got information about sildenafil primarily from their friends. Similar results were reported in other studies in which friends, the internet, and sex shops were the primary source of information (6). Information sought from sources other than health professionals could lead to unexpected consequences that could be directly related to the medication (sildenafil) itself, such as priapism or due to an interaction between sildenafil and other medications the user may take or comorbid conditions he/she may have (4, 5, 13–18). The recreational use of sildenafil was also associated with vision changes (24).

A remarkable issue evident in the current study and other similar studies was the level of acquisition of sildenafil without a medical prescription. In the present study, most of the respondents get sildenafil without a prescription as an OTC medication. Similar findings were reported in Ethiopia and a web-based observational study conducted in the United Kingdom, Germany, and Italy (1, 9). The latter revealed that embarrassment to speak to a physician and the perception that this would be the cheapest way to get the medicine was the major predictive factors for obtaining PDE5Is without prior health care professional consultation (9). The non-prescription availability of PDE5Is could lead to the inappropriate utilization of the medication and an increased rate of adverse effects as well as increased probability of addiction to the drug (14).

The most common reasons to use sildenafil in the present study were to increase erectile sensation and penile rigidity. Similar reasons were reported in a study conducted in Argentina in which a desire to increase penile rigidity, coital frequency, erection duration and to prevent sexual failure were the common reasons for PDE5Is use (6).

In the present study, some of the respondents reported the use of alcohol and multiple illicit drugs with sildenafil in which more than half of the respondents reported they use alcohol 63.1% followed by cigarettes 36.9% and khat (23.1%). The result was consistent with a previously conducted similar study in Ethiopia (1). In Ethiopia alcohol, khat (Catha edulis), and tobacco are the most frequently used recreational substances and the rate of substance use is increasing, particularly in the younger population (12, 25). The use of these substances along with sildenafil could lead to adverse consequences due to pharmacokinetic or pharmacodynamic interactions and could also lead to death at some point. Evidence showed that significant interactions can occur between illicit drugs and sildenafil (26). Cigarette and/or cannabis smoking was also found to alter the exposure of sildenafil and increase its plasma concentration (27).

The most common side-effects associated with the use of sildenafil as reported by the study participants were dizziness and headache. From the extensive clinical trials, the most commonly reported adverse events in patients treated with sildenafil were headache and flushing (2). These symptoms are evident since sildenafil alone can cause mean peak reductions in systolic/diastolic blood pressure of 10/7 mm Hg that are not dose-related (4, 5). This reduction in blood pressure could lead to hypotension which may be the cause for the symptoms of headache, dizziness, and flushing.

### Limitation of the Study

Since the study was a cross-sectional study based on the honesty of the participants, there could be respondent bias as the respondents may not tell their genuine behavior to some of the questions due to the sensitivity of the topic. Another limitation could be the low number of study participants as this may limit the probability of getting some hidden behaviors and which may result in sparse data bias. However, despite these limitations, the current study was conducted in new areas with no other local study before. It signals future researchers on this area and the stakeholders to take legal measures on the issue of sildenafil selling without a prescription and its rational utilization.

## Conclusion

Most men who use sildenafil citrate do so for recreational purposes, and use of sildenafil citrate for 2 years or less was associated with recreational use. The availability of sildenafil without a prescription paper and lack of concomitant monitoring of its utilization were some of the main contributing factors for its misuse. Consumption of sildenafil with illicit drugs was also reported. There should be a collaborative effort among pharmacists, health professionals, and policymakers to improve the rational use of sildenafil citrate.

### Recommendation

Health care professionals and especially pharmacists should counsel sildenafil users on how and when to use the medication and the common side effects associated with the drug. Users should also be aware of the impact of using sildenafil concomitantly with other illicit drugs and advised to use it when it is only necessary to avoid its addiction and minimize adverse effects. The regulatory body should control the availability of sildenafil and other PDE5Is in places other than pharmacies, monitor the sale of prescription-only medications on an OTC basis, and monitor their utilization.

## Data Availability Statement

The raw data supporting the conclusions of this article will be made available by the authors, without undue reservation.

## Ethics Statement

The studies involving human participants were reviewed and approved by Ethical Review Committee of the School of Pharmacy, University of Gondar, Ethiopia with an approval number of SoP 826/09. The patients/participants provided their written informed consent to participate in this study.

## Author Contributions

All authors had contributed to the data analysis, drafting, or revising the article. They gave the final approval of the manuscript to be published and agreed to be accountable for all aspects of the work.

## Conflict of Interest

The authors declare that the research was conducted in the absence of any commercial or financial relationships that could be construed as a potential conflict of interest.

## Publisher's Note

All claims expressed in this article are solely those of the authors and do not necessarily represent those of their affiliated organizations, or those of the publisher, the editors and the reviewers. Any product that may be evaluated in this article, or claim that may be made by its manufacturer, is not guaranteed or endorsed by the publisher.
